# Effects of sintering temperature on surface morphology/microstructure, *in vitro* degradability, mineralization and osteoblast response to magnesium phosphate as biomedical material

**DOI:** 10.1038/s41598-017-00905-2

**Published:** 2017-04-11

**Authors:** Zhiwei Wang, Yuhai Ma, Jie Wei, Xiao Chen, Liehu Cao, Weizong Weng, Quan Li, Han Guo, Jiacan Su

**Affiliations:** 1 Department of Orthopaedics, Changhai Hospital, Second Military Medical University, Shanghai, 200433 China; 2 Department of Orthopaedics, Zhejiang Provincial Armed Police Corps Hospital, Hangzhou City, Zhejiang Province 310051 P.R. China; 3 Key Laboratory for Ultrafine Materials of Ministry of Education, East China University of Science and Technology, Shanghai, 200237 P.R. China; 4 Shanghai Synchrotron Radiation Facility, Shanghai Institute of Applied Physics, Chinese Academy of Sciences, Shanghai, 201800 P.R. China

## Abstract

Magnesium phosphate (MP) was fabricated using a chemical precipitation method, and the biological performances of MP sintered at different temperatures as a biomedical material was investigated. The results indicated that the densification and crystallinity of MP increased as the sintering temperature increased. As the sintering temperature increased, the degradability of MP in PBS decreased, and the mineralization ability in SBF significantly increased. In addition, the MP sintered at 800 °C (MP8) possessed the lowest degradability and highest mineralization ability. Moreover, the positive response of MG63 cells to MP significantly increased as the sintering temperature increased, and MP8 significantly promoted the cell spreading, proliferation, differentiation and expressions of osteogenic differentiation-related genes. Faster degradation of MP0 resulted in higher pH environments and ion concentrations, which led to negative responses to osteoblasts. However, the appropriate degradation of MP8 resulted in suitable pH environments and ion concentrations, which led to positive responses to osteoblasts. This study demonstrated that the sintering temperature substantially affected the surface morphology/microstructure, degradability and mineralization, and osteoblasts response to magnesium phosphate.

## Introduction

In the past few decades, some bioactive materials containing phosphate (P) element have been reported including calcium phosphate (Ca-P) biomaterials, such as hydroxyapatite, tricalcium phosphate and calcium phosphate cements, and P-containing bioglasses/bioceramics/cements^1–3^. These P-containing bioactive materials that exhibit excellent biocompatibility and bioactivity have been applied to bone repair and used as a bone substitute in the form of particles, blocks and coatings^4, 5^. In addition, magnesium (Mg) based biomaterials, such as Mg-associated alloys, Mg-containing bioactive glasses/bioceramics and coatings, and Mg-substituted/Mg-based calcium phosphate cements, have also been reported due to their good biocompatibility and bioactivity, etc.^6–9^. Magnesium ions (Mg^2+^) play critical roles in bone remodeling and skeletal tissue development in the human body^10^. Moreover, Mg^2+^ may improve bone mineral density and affect bone fragility, and a lack of Mg^2+^ affects all stages of skeletal metabolism, leading to slower bone growth, and osteoporosis^11, 12^.

Previous studies investigated several Mg-P based cements for bone repair, which were prepared by mixing magnesium oxide (MgO) with sodium dihydrogen phosphate (NaH_2_PO_4_), ammonium dihydrogen phosphate (NH_4_H_2_PO_4_), and calcium dihydrogen phosphate (Ca(H_2_PO_4_)_2_) to form cements powders^13–15^. These cement powders were mixed with water to form pastes and converted to magnesium calcium phosphate (MgNaPO_4_), magnesium ammonium phosphate (MgNH_4_PO_4_), and magnesium calcium phosphate (Mg(CaPO_4_)_2_), which exhibited good *in vitro* and *in vivo* biocompatibility, bioactivity as well as degradability^15–17^. Furthermore, electrochemically assisted deposition has been employed to produce ceramic coatings based on MgNH_4_PO_4_ on corundum-blasted titanium surfaces^18^.

In recent years, magnesium phosphate (Mg-P) materials have been investigated for bone tissue engineering applications due to their biocompatibility and biodegradability. Lee J. *et al*. developed a 3D porous magnesium phosphate (Mg-P) scaffold with a high drug load/release efficiency for use in hard tissue regeneration using a paste extruding deposition (PED) system and cement chemistry^19^. Meininger S. *et al*. prepared Sr-substituted Mg_3_(PO_4_) based biodegradable scaffolds using three-dimensional powder printing, followed by high temperature sintering and/or chemical conversion for patient-specific implants^20^. The amorphous magnesium phosphate in a nanospherical form from an aqueous solution containing Mg^2+^ and HPO_4_^2−^/PO_4_^3−^ was produced using a novel microwave-assisted approach, and this material self-assembled into mature Mg-P materials and supported cell proliferation^21^. Fibrous bionanocomposites consisting of amorphous magnesium phosphate nanospheres and polylactic acid were fabricated by electrospinning^22^. Mg and P containing biomaterials are expected to possess good biocompatibility, degradability and bioactivity, which are required for new bioactive materials for bone repair. Therefore, in this study, magnesium phosphate (MP) was fabricated using the chemical precipitation method and sintered at different temperatures, and the effects of the sintering temperature on the surface morphology/microstructure, *in vitro* degradability and mineralization, and osteoblast response to magnesium phosphate as a biomedical materials for bone repair were investigated.

## Results

### Characterizations of as-prepared MP0

**Figure 1:**
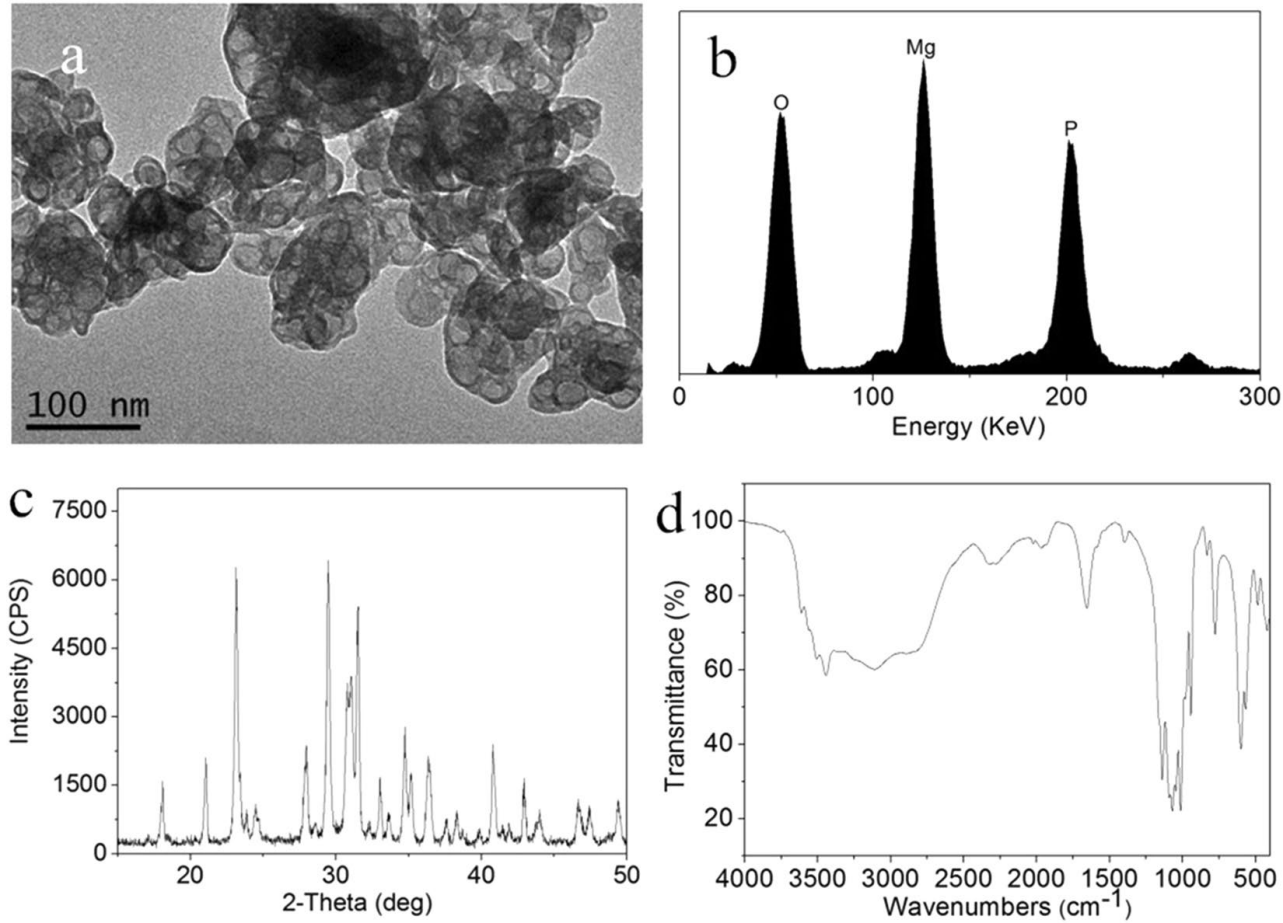
TEM image (**a**) and EDS (**b**), XRD (**c**) and FTIR (**d**) results for the as-prepared MP0.

The TEM image of the surface morphology of the as-prepared MP0 is shown in Fig. 1. The particle size of MP0 ranged from 20 nm to 50 nm.

Figure 1b shows the EDS of MP0, which contained Mg, P and O elements. Figure 1c shows the XRD of MP0. The peaks at 2θ = 13.5°, 21.0°, 23.1°, 24.2°, 27.9°, 29.3°, 31.4°, 33°, 35.1°, 36.4°, 40.9° and 43.1° correspond to MP0^23, 24^. The results confirmed that the as-prepared materials consisted of an MP phase, which existed as Mg_3_(PO_4_)_2_·5H_2_O, and no additional phases were identified. Figure 1d shows the IR spectrum for MP0. The absorption bands at 587 cm^−1^ and 1071 cm^−1^ were due to PO_4_^3−^, and the broad band at 3472 cm^−1^ corresponds to absorbed water.

### SEM, XRD and IR analyses of MP sintered for different time periods

**Figure 2:**
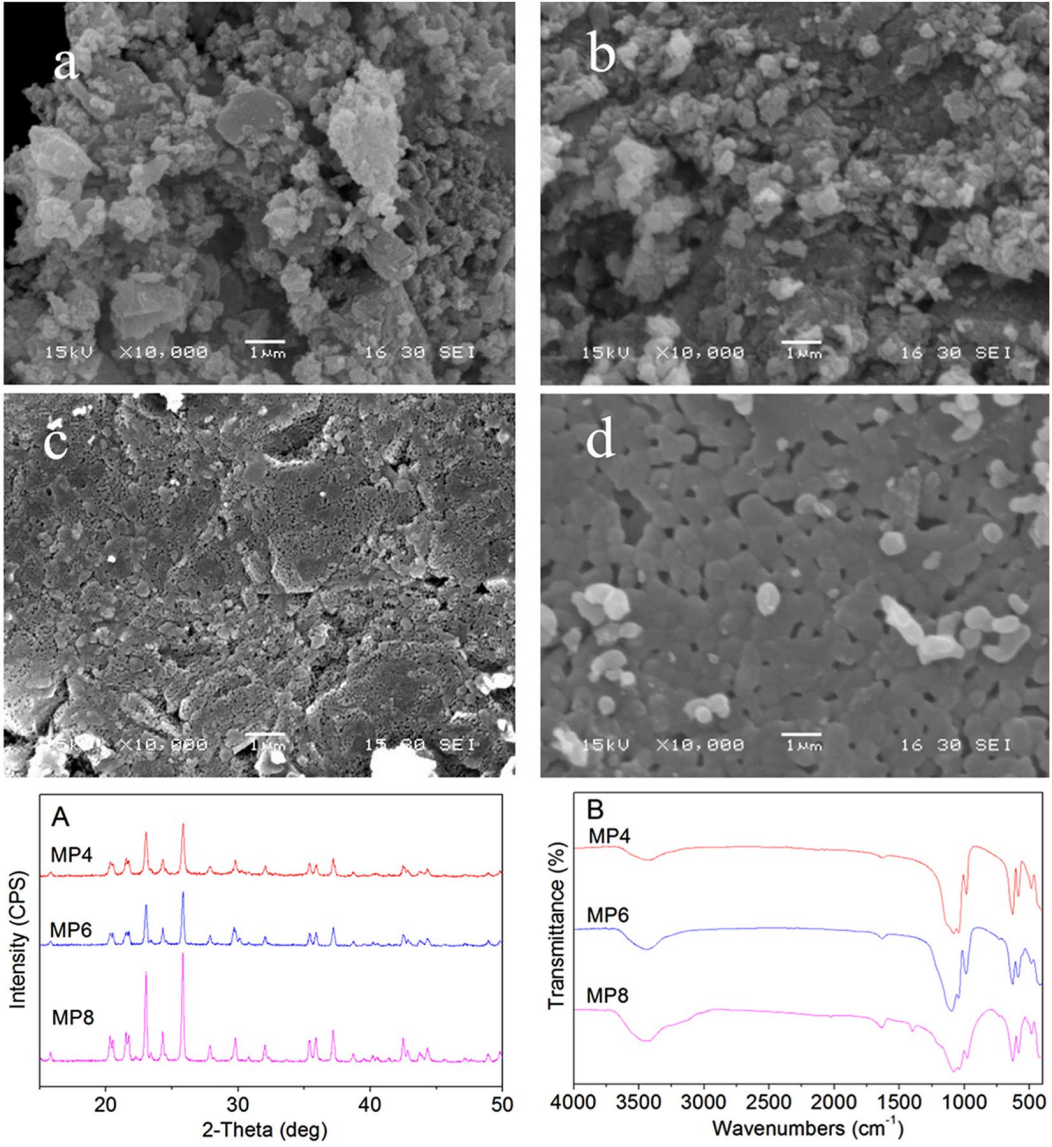
SEM images of the surface morphology/microstructure of MP0 (**a**), MP4 (**b**), MP6 (**c**) and MP8 (**d**) as well as the XRD (**A**) and IR (**B**) results for MP4, MP6 and MP8 after sintering MP0 at 400 °C, 600 °C and 800 °C.

Figure 2 shows the SEM images of the surface morphology/microstructure of MP0, MP4, MP6 and MP8. The surfaces of MP0 and MP4 are loose structures with many irregular particles (amorphous form) on the surfaces. However, the surfaces of MP6 and MP8 possessed dense structures containing many crystalline particles. The results indicated that the surface/microstructure of MP became denser as the sintering temperature increased.

Figure 2A shows the X-ray diffraction patterns of the as-prepared MP0 sintered at 400 °C, 600 °C and 800 °C. The peaks at 2θ = 20.9°, 21.1°, 22.5°, 24.3°, 25.6°, 27.5°, 29.4°, 31.5°, 33°, 35.1°, 35.4°, 36.7, 40.8° and 43.0° were due to MP, and no obvious changes in the peak positions were observed for the MP sintered at different temperatures (i.e., MP4, MP6, and MP8) compared to those for MP0. Furthermore, the crystallinity of MP increased with the sintering temperature.

Figure 2B shows the IR spectra of the as-prepared MP0 sintered at 400 °C, 600 °C and 800 °C. The absorption bands at 587 cm^−1^ and 1071 cm^−1^ were due to PO_4_^3−^, and the broad band at 3472 cm^−1^ was attributed to absorbed water. The results indicated that the absorption bands for the MP sintered at different temperatures (i.e., MP4, MP6, and MP8) did not change compared to those for MP0.

### Degradability and pH change in PBS

**Figure 3:**
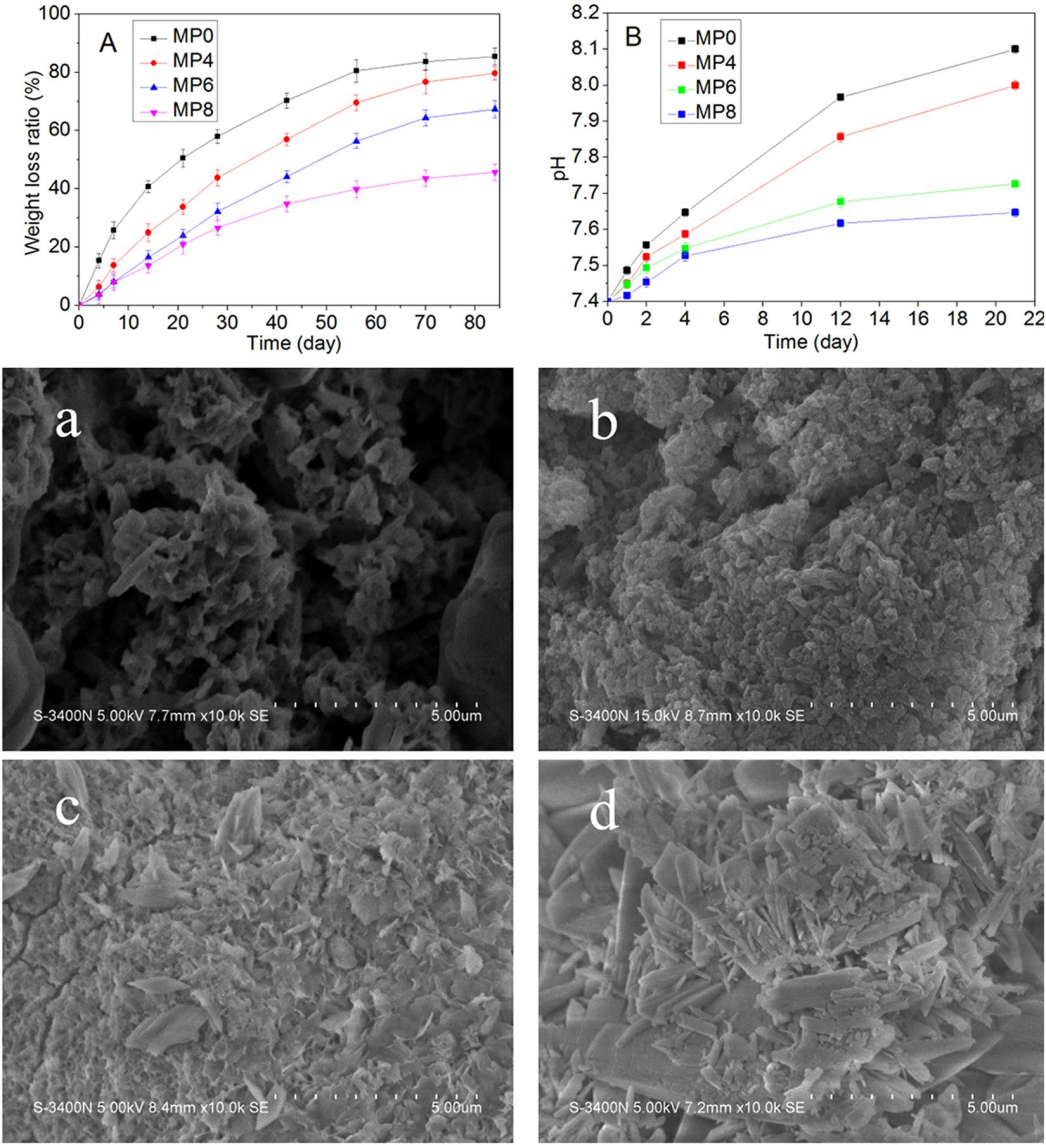
Weight loss (**A**) and pH change in the solutions (**B**) after MP0, MP4, MP6 and MP8 were immersed in a PBS solution for different time periods. In addition, the SEM images show the surface morphology after MP0 (**a**), MP4 (**b**), MP6 (**c**) and MP8 (**d**) were soaked in a PBS solution for 3 weeks.

The weight loss of MP immersed in phosphate buffer saline (PBS) for different time periods is shown in Fig. 3A. The weight loss of MP increased with the time and sintering temperature. After immersion into PBS for 12 weeks, the weight loss ratios of MP0, MP4, MP6 and MP8 were 85.46 w%, 79.65 w%, 67.34 w% and 45.67 w%, respectively.

The pH change of the solution was monitored after MP was soaked in PBS for different time periods, and the results are shown in Fig. 3B. The pH of MP increased with time and decreased with the sintering temperature. After immersion for 4 weeks, the pH values for MP0, MP4, MP6 and MP8 were 8.1, 8.0, 7.7 and 7.6, respectively.

Figuure 3 shows the surface morphology/microstructure of MP after immersion in the PBS solution for 4 weeks. MP0 and MP4 were degraded, and the surfaces of MP0 and MP4 contained loose structure with many micropores. However, the surfaces of MP6 and MP8 possessed dense structures with many crystalline substances.

### Mineralization of MP in SBF

**Figure 4:**
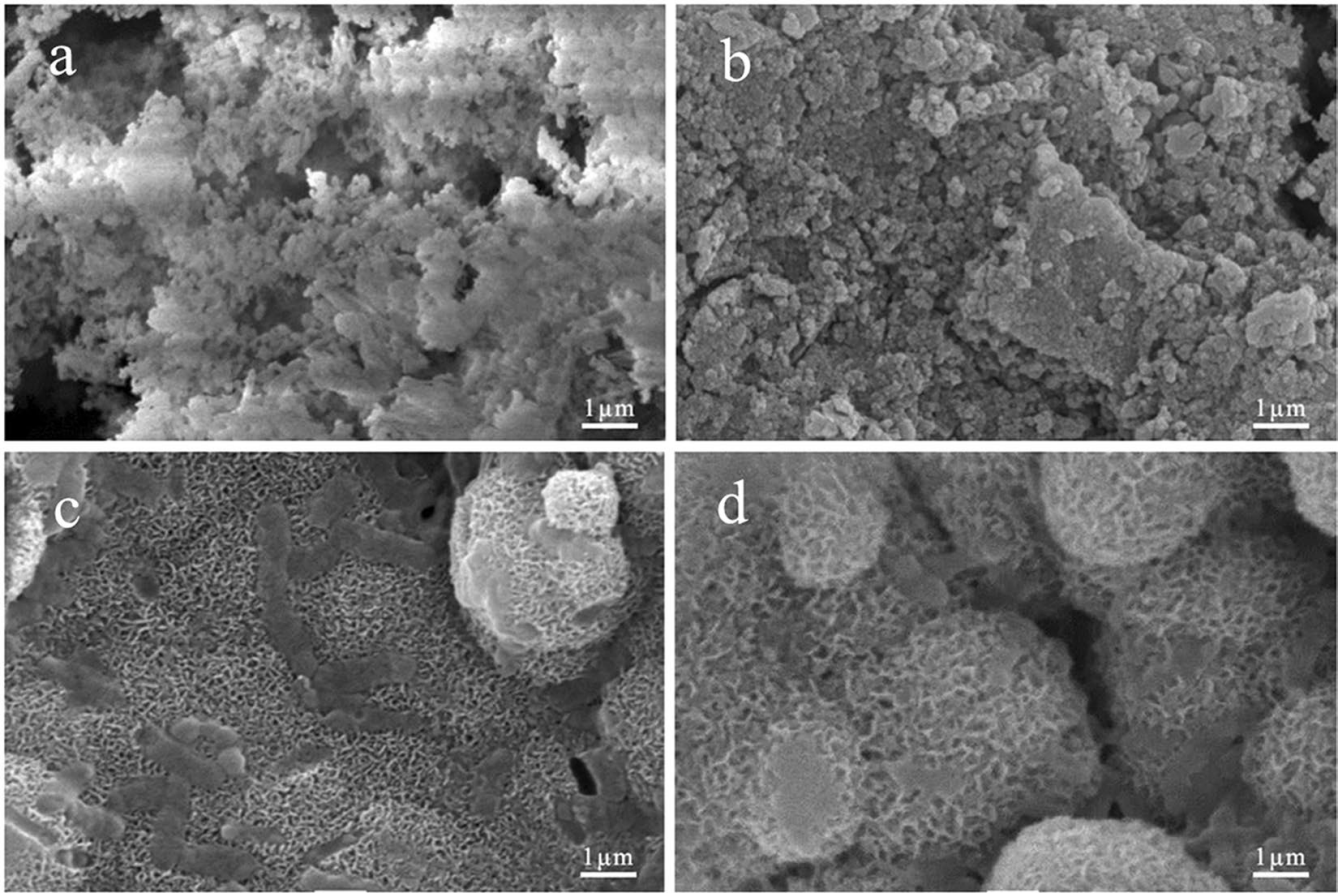
SEM images of MP0 (**a**), MP4 (**b**), MP6 (**c**) and MP8 (**d**) after soaking in SBF for 7 days.

The *in vitro* mineralization of MP in simulated body fluid (SBF) was evaluated after MP was soaked in SBF for different time periods^25^. Figure 4 shows the SEM images of the surface morphology of MP0, MP4, MP6 and MP8 after soaking in SBF for 7 days. No apatite formation was observed on the MP0 and MP4 surfaces. However, a layer of ball-like particles, which is characteristic of apatite, appeared on the MP6 and MP8 surfaces.

**Figure 5:**
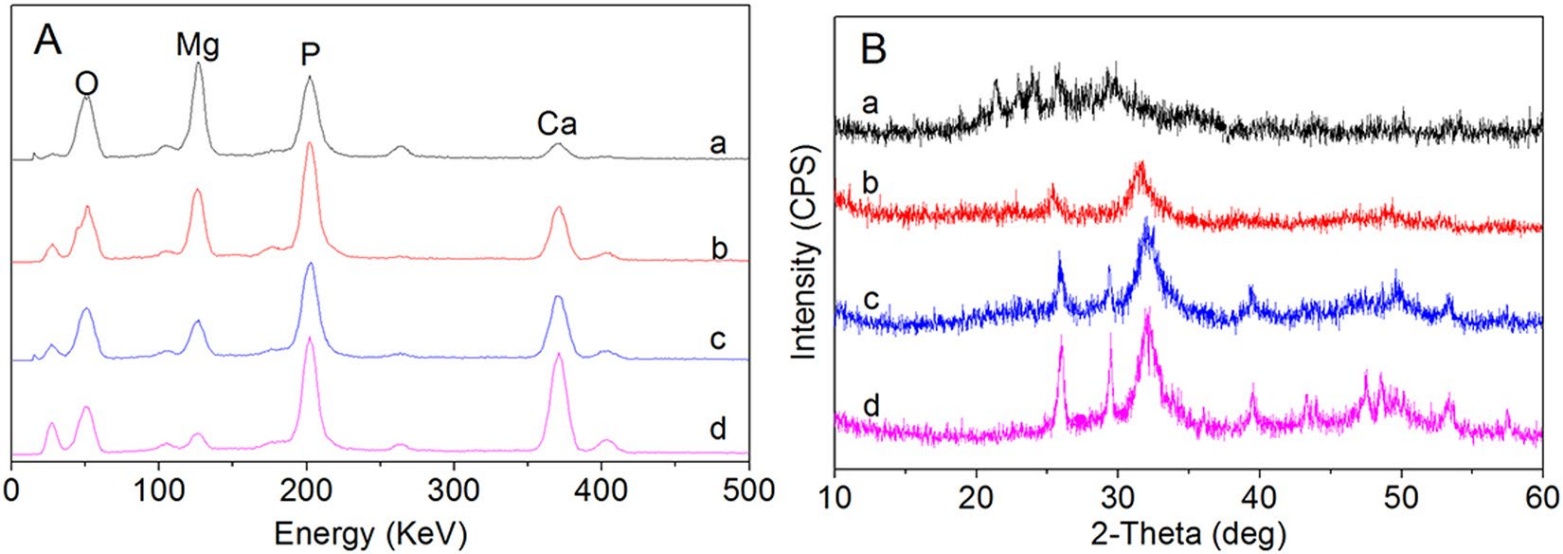
EDS and XRD results for MP0 (**a**), MP4 (**b**), MP6 (**c**) and MP8 (**d**) after soaking in SBF for 7 days.

Figure 5A shows the EDS results for MP0, MP4, MP6 and MP8 after soaking in SBF for 7 days. Mg, P and Ca peaks appeared. As the sintering temperature increased, the Mg peaks decreased, and the Ca peaks increased, indicating that Ca-P-containing particles were deposited on the surfaces of MP6 and MP8.

Figure 5B shows the X-ray diffraction results for MP0, MP4, MP6 and MP8 after soaking in SBF for 7 days. The peaks at 2*θ* = 25.8°, 29.6°, 31.9°, 39.7° and 48.6°, which correspond to the apatite in MP6 and MP8, demonstrated that apatite formed on the MP surfaces as the sintering temperature increased.

**Figure 6:**
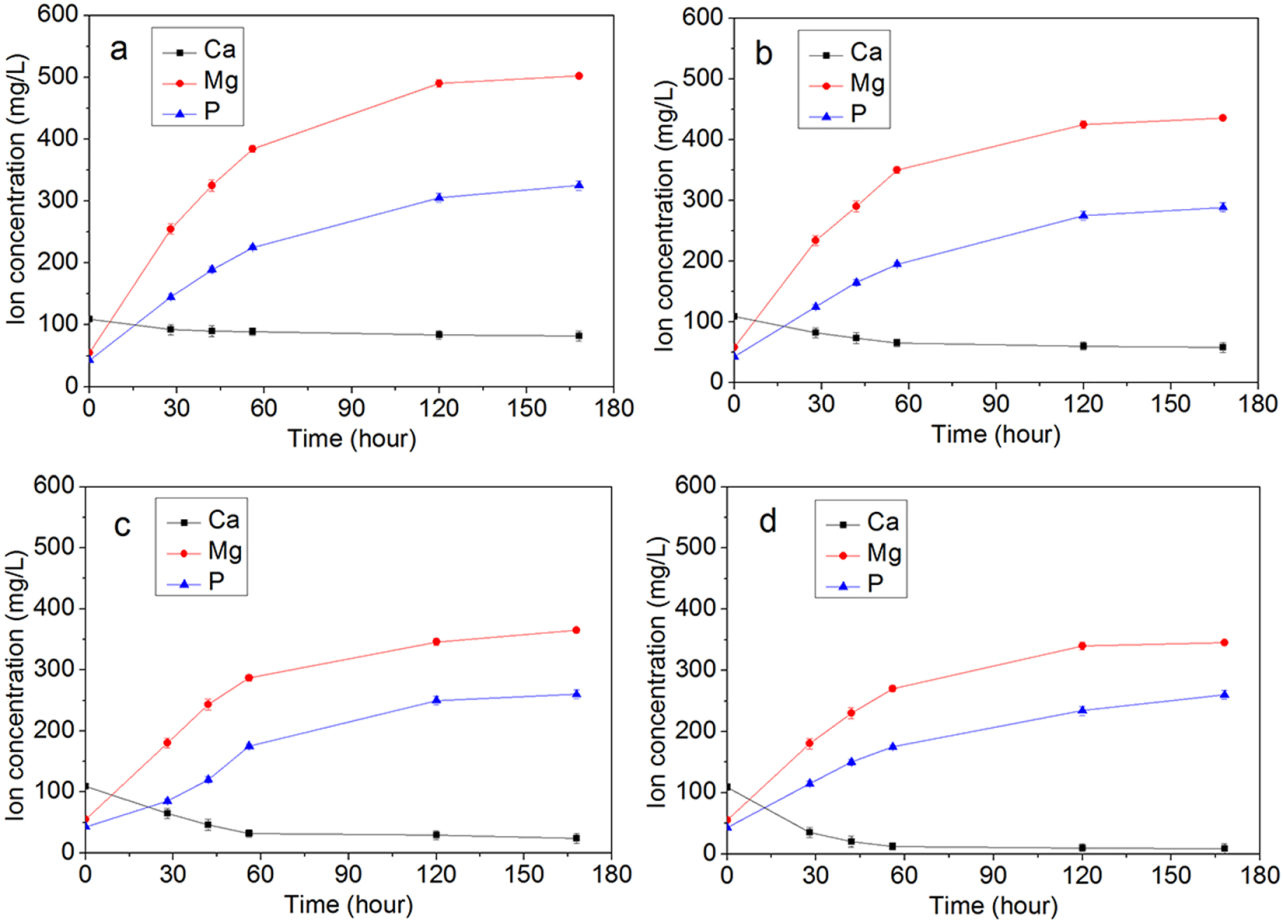
Changes in the Ca, Mg and P ion concentration in the solutions after MP0 (**a**), MP4 (**b**), MP6 (**c**) and MP8 (**d**) were soaked in SBF for different time periods.

Figure 6 reveals the changes in the ions concentrations of Ca, Mg and P in solution after MP0, MP4, MP6 and MP8 were soaked in SBF for different time periods. Both the Mg and P ion concentrations in SBF increased rapidly during the first 3 days and slower until day 7. In addition, the Ca ion concentration decreased over time. After the samples were soaked in SBF for 7 days, the Mg, P, and Ca ion concentrations for MP0, MP4, MP6 and MP8 in SBF are shown in Table S2. The results indicated that the Mg, P and Ca ion concentrations in SBF decreased as the sintering temperature increased (MP0 < MP4 < MP6 < MP8).

### Cell proliferation, morphology and alkaline phosphatase (ALP) activity

**Figure 7:**
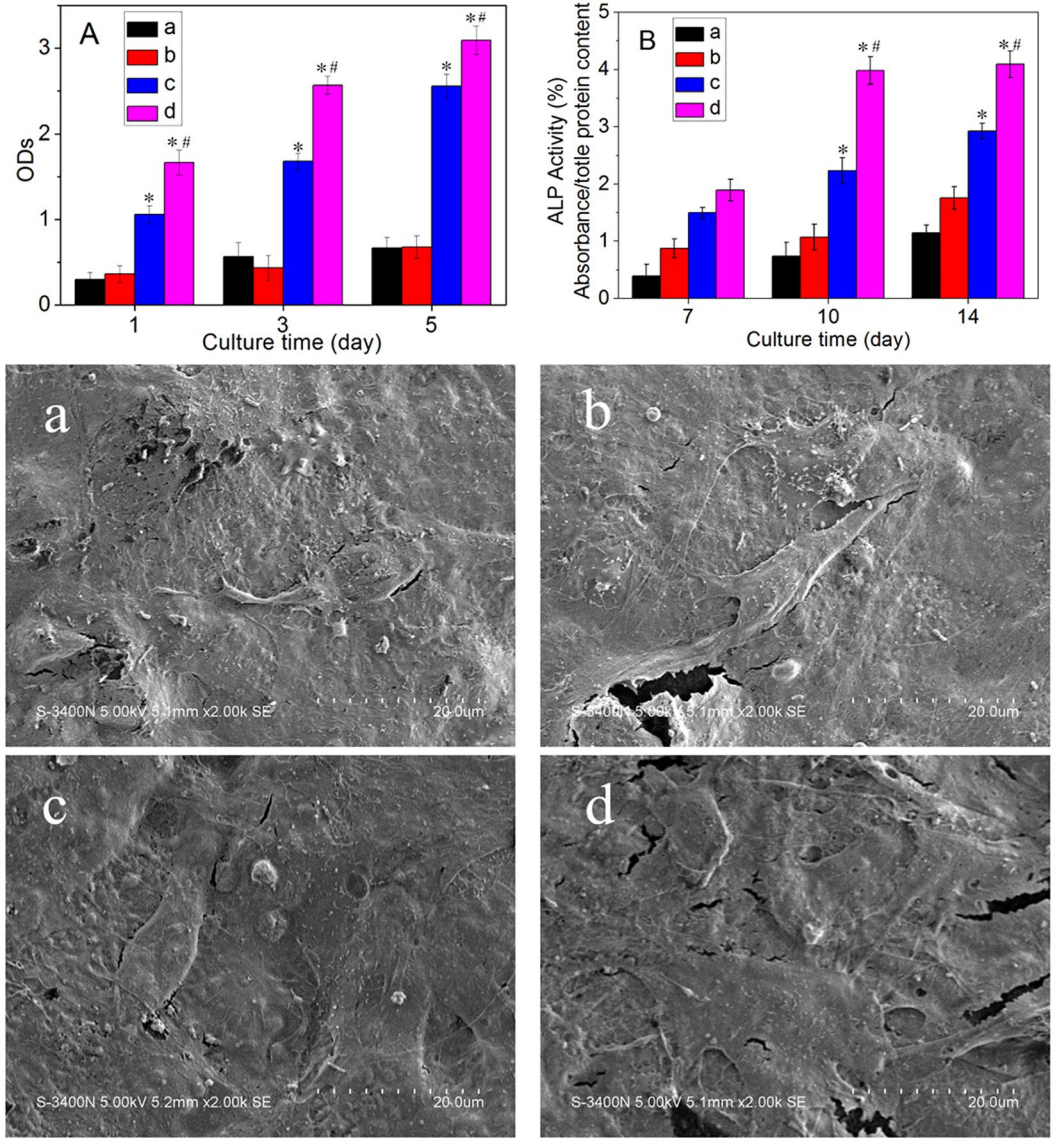
MTT essay (**A**) and ALP activity (**B**) of MG63 cells cultured on MP0 (**a**), MP4 (**b**), MP6 (**c**) and MP8 (**d**) for different time periods. The SEM images show the cell morphology of MG63 cells cultured on MP0 (**a**), MP4 (**b**), MP6 (**c**) and MP8 (**d**) for 5 days. “*” denotes significant differences compared to MP0 and MP4,“#” denotes significant differences compared to MP6.

Figure 7A shows the proliferation of MG63 cells on MP measured using the cell counting kit-8 (CCK-8) assay. The ODs of the relative proliferation ratios of the cells on MP6 and MP8 significantly increased with culture time. However, no increase was ovserved on MP0 and MP4. In addition, the relative proliferation ratios of the cells on MP6 and MP8 were significantly higher than those on MP0 and MP4 at day 1, 3 and 5, and MP8 exhibited an even higher ratio than MP6.

Figure 7B shows the alkaline phosphatase (ALP) activity of MG63 cells on MP at days 7, 10 and 14. The ALP activities of the cells on MP8 were significantly high at days 10 and 14 compared with day 7. In addition, no change was observed at 10 and 14 days.The ALP activities of cells on MP8 were significantly higher than those on MP0, MP4 and MP6 at days 10 and 14, and the activity on MP6 was higher than those on MP0 and MP4. Moreover, no obvious changes in the ALP activities were observed for MP0 and MP4.

Figure 7 shows the SEM images of the cell morphology of the MG63 cells grown on MP (i.e., MP0, MP4, MP6 and MP8) for 5 days. Only a few cells were attached to the MP4 and MP0 surfaces. However, the cells exhibited a typical fibroblastic morphology with more cytoplasmic extensions and better filopodial attachments on the MP6 and MP8 surfaces than on the MP4 and MP0 surfaces. In addition, MP8 exhibited better cell spreading than MP6.

### Osteogenic differentiation-related genes expression

**Figure 8:**
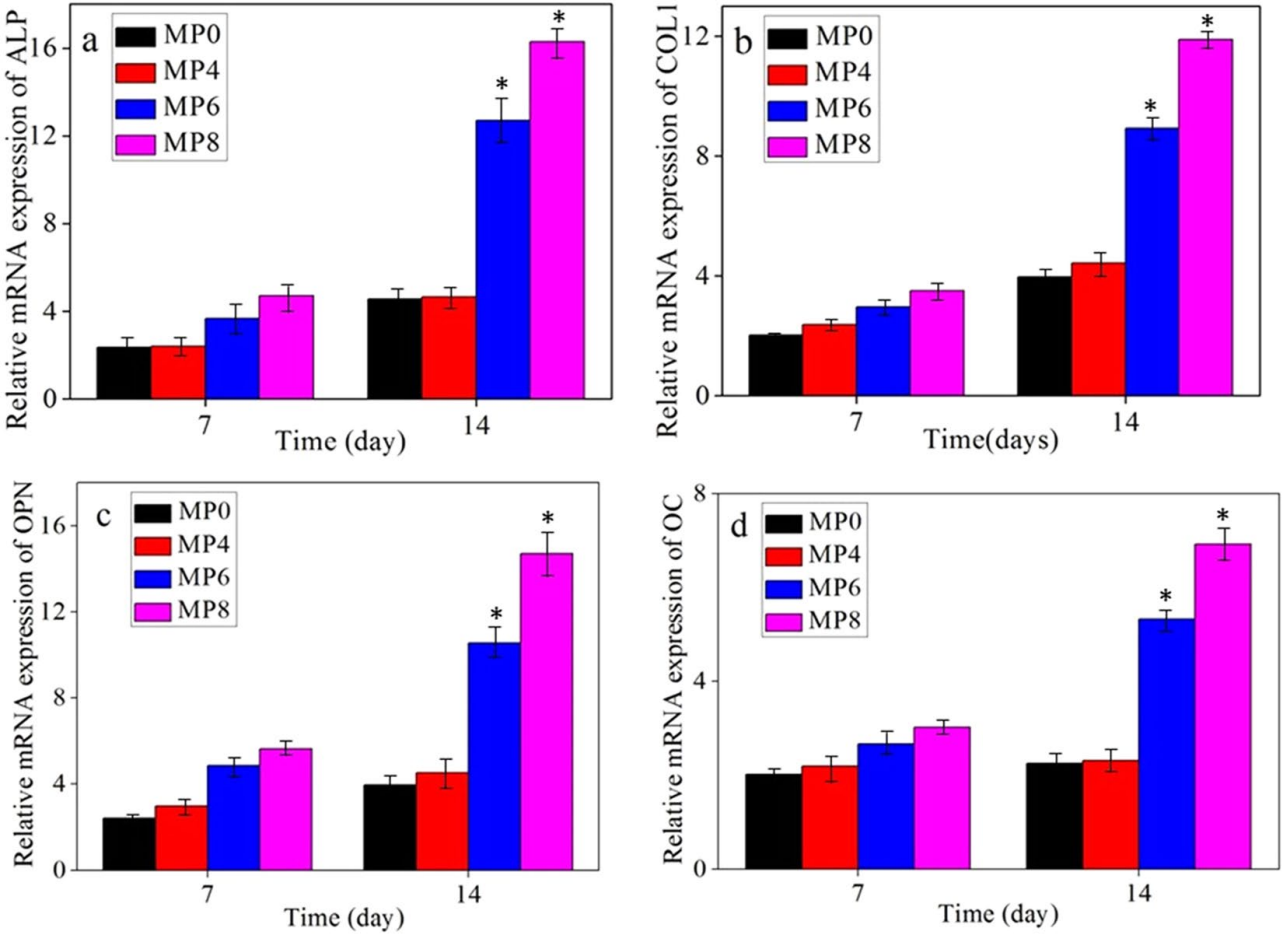
Relative mRNA expression of osteogenic differentiation-related genes: ALP (**a**), COL-1 (**b**), OPN (**c**) and OC (**d**) for MG63 cells grown on MP0, MP4, MP6 and MP8.

Figure 8 shows the mRNA expression of osteogenic differentiation-related genes determined by real-time PCR. At day 7, the ALP, COL1, OPN, and OC mRNA of the cells on MP8 exhibited high expressions compared those on MP0, MP4 and MP6, and no difference was observed for MP0, MP4 and MP6. At day 14, the ALP, COL1, OPN, and OC mRNA of the cells on MP8 and MP6 exhibited significantly higher expressions than those on MP0 and MP4. In addition, MP6 was higher than MP0 and MP4.

## Discussions

In recent years, magnesium (Mg) based bioactive inorganic materials, such as Mg-containing bioactive glasses/bioceramics and coatings as well as Mg-based/Mg-substituted calcium phosphate bone cement, have received increasing attention for use in bone repair applications^26^. In this study, magnesium phosphate as a biomedical materials for bone regeneration was fabricated, and the effects of the sintering temperature on the surface morphology/microstructure, *in vitro* mineralization and degradability, and cell response to MP were investigated. The results indicated that the as-fabricated MP0 particles with a size ranging from 50 nm to 100 nm possessed a weak crystalline structure (amorphous form), which existed as Mg_3_(PO_4_)_2_·5H_2_O. After sintering at different temperatures (i.e., 400 °C, 600 °C and 800 °C), the MP crystallinity (MP4, MP6, and MP8) increased with the sintering temperature. The MP0 and MP4 surfaces contained loose structures with many irregular particles (amorphous form), and the surfaces of MP6 and MP8 possessed dense structures containing many crystalline particles, indicating that the surface/microstructure of MP became denser as the sintering temperature increased.

For the design and fabrication of a biomaterial for bone regeneration, suitable degradability is desired (approximately 3 months)^27^. In this study, the degradability of MP sintered at different temperatures was determined by testing the weight loss ratios of samples after being soaked in PBS for different time periods. The results revealed that the degradability of MP in PBS decreased with the sintering temperature (MP0 < MP4 < MP6 < MP8). The MP0 and MP4 degraded in PBS, and the surfaces of MP0 and MP4 possessed a loose structure with many micropores, which may increase the surface area of the samples, enlarged the contact area of the samples with solution, and accelerate the dissolution process. However, the surfaces of MP8 and MP6 possessed a dense structure, indicating gradual degradation of the samples surfaces. The results indicated that the sintering temperature had a significantly effect on the degradability of MP. A high sintering temperature enhanced the crystallinity of the MP, decreasing the dissolution of MP in water.

For degradable biomaterials, the dissolution/release of acidic or alkaline degradation by-products could lead to *in vivo* inflammatory/negative responses from cells/tissues. Some approaches have attempted to stabilize the pH in the physiological environment to avoid a significant decrease or increase during the degradation of the biomaterials^28^. In this study, the pH value in PBS containing MP increased with time, indicating that some alkaline products, such as Mg^2+^, may have been produced during MP degradation. Moreover, the pH values for MP0, MP4, MP6 and MP8, which was soaked in PBS for 4 weeks, were 8.1, 8.0, 7.7 and 7.6, indicating that the pH value for MP decreased as the sintering temperature increased (MP0 > MP4 > MP6 > MP8). Therefore, the sintering temperature of MP may control the pH increase. Clearly, the dissolution of MP8 resulted in a weak alkaline environment (pH = 7.6) that was close to the pH of the physiological environment (pH = 7.4). However, MP0 resulted in a more alkaline environment (pH = 8.0). A previous study demonstrated that a weak alkaline condition could provide a better micro-environment for cell proliferation and subsequent differentiation^29^. Therefore, MP8 may provide a suitable micro-environment for cell proliferation, differentiation and new bone tissue formation *in vivo*.

The ability to induce the apatite formation on the surface of a biomaterial exposed to relevant biological fluids, such as SBF, can elucidate the *in vitro* bioactivity and predict the ability for bone regeneration or the generation of interfacial bonding with bone tissue *in vivo* ^30^. In this study, the results indicated that no apatite was formed on the MP0 and MP4 surfaces. However, a layer of ball-like apatite particles was observed on the MP6 and MP8 surfaces after soaking in SBF for 7 days. The EDS results indicated that the Ca peak significantly increased on the MP surfaces as the sintering temperature increased (MP0 < MP4 < MP6 < MP8). In addition, the Mg peak decreased. Therefore, Ca-P substances were deposited on the samples surfaces, which increased as the sintering temperature increased. Furthermore, the XRD results further demonstrated that the deposits consisted of apatites, which increased as the sintering temperature increased (apatite-layer appeared on the MP6 and MP8 surfaces). The changes in the Ca, Mg and P ion concentrations in the solution after MP0, MP4, MP6 and MP8 were soaked in SBF for different time periods were investigated. The results indicated that Mg and P ion concentrations in SBF containing MP increased with time but the Ca ion concentrations decreased, indicating the dissolution of MP and the deposition of apatite on the material surface. Moreover, the Ca ion concentration decreased as the sintering temperature of MP increased (MP0 < MP4 < MP6 < MP8). Therefore, more apatite was deposited on MP8 than the other samples, which is in agreement with the results of SEM, EDS and XRD results.

The apatite that precipitates on bioactive materials surfaces involves a process consisting of dissolutions (ions release) and deposition (Ca-P substances)^31^. In this study, the dissolution of MP0 and MP4 was faster than the deposition of Ca-P substances due to the high solubility of MP0 and MP4. Therefore, the ability of apatite to deposit on the MP0 and MP4 surfaces in SBF was poor. However, the solubility of MP6 and MP8 decreased as the sintering temperature increased due to the increase in crystallinity. Therefore, the apatite could be deposit on the surfaces.

After the MG63 cells were cultured on the samples, only a few cells grew on the MP4 and MP0 surfaces. However, the cells on the MP6 and MP8 surfaces exhibited a typical fibroblastic morphology with more cytoplasmic extensions as well as better filopodial attachment and growth than MP4 and MP0. In addition, MP8 was better than MP6. The proliferation of MG63 cells on MP6 and MP8 significantly increased with time (no increases were observed on MP0 and MP4) and was significantly higher than that on MP0 and MP4. In addition, MP8 was better than MP6. The results indicated that MP6 and MP8, which exhibited good cytocompatibility and bioactivity, enhanced cell attachment, spreading, growth and proliferation. The ALP activities of the MG63 cells for MP8 were significantly higher than those for MP0, MP4 and MP6 at 10 and 14 days, and the activities for MP6 were higher than those for MP0 and MP4, indicating that MP8 promoted cell differentiation. The ALP, COL1, OPN, and OC mRNA of MG63 cells on MP8 exhibited significantly higher expressions than those on MP0, MP4 and MP6, indicating that MP8 significantly improved the expressions of osteogenic differentiation-related genes.

Mg dissolution products from bioactive glasses/ceramics could stimulate proliferation, differentiation and expression of osteogenic-related genes during the incubation of osteoblasts^32^. Furthermore, the P ions released from the bioactive materials play an important role in stimulating osteoblast proliferation and osteoblastic differentiation^33^. Moreover, the apatite formed on the bioactive materials surfaces facilitated osteoblasts attachment, proliferation and differentiation^34^. Our results indicate that the degradability of MP affects the biological response of osteoblasts including cell attachment, proliferation, differentiation, and osteogenic differentiation-related genes expression. More rapid degradation of MP (MP0 and MP4) resulted in higher pH environments and higher ion concentrations (Mg and P ions), which led to negative responses to osteoblasts. However, suitable degradation of MP (MP6 and MP8) resulted in suitable pH environments and ion concentrations, which led to positive responses to osteoblasts. Therefore, the enhancement of the biological responses of MP8 to osteoblasts may be due to suitable degradability, good bioactivity (apatite formation ability), weak alkaline micro-environments and appropriate Mg and P ions concentrations, which promoted attachment, proliferation, differentiation, and osteogenic differentiation-related gene expression of osteoblasts. MP8, which possesses suitable degradability, good bioactivity and excellent cytocompatibility, has the potential for use as a new type of bone implant material.

## Conclusions

Magnesium phosphate (MP) sintered at different temperature were fabricated for use as biomedical materials. The results indicated that the densification and crystallinity of MP increased as the sintering temperature increased. In addition, the degradability of MP decreased and the bioactivity increased significantly as the sintering temperature increased. MP8 exhibited the lowest degradability and highest bioactivity. Moreover, the positive responses of MG63 cells to MP increased significantly as the sintering temperature increased, and MP8 significantly promoted cell spreading, proliferation, differentiation and expressions of osteogenic differentiation-related genes.

The sintering temperature significantly affected the surface morphology/microstructure, *in vitro* mineralization, degradability and cell responses to magnesium phosphate, and the enhancement of the positive responses of osteoblasts to MP8 was due to suitable degradability, good bioactivity, weak alkaline micro-environments and appropriate Mg and P ions concentrations. Therefore, MP8, which possesses suitable degradability, good bioactivity and excellent cytocompatibility.

## Material and Methods

### Fabrication of magnesium phosphate (MP)

Magnesium phosphate (MP) was synthesized using chemical precipitation. Magnesium nitrate [Mg(NO_3_)_2_·6H_2_O, Sinopharm Chemical Reagent Co., Ltd.] and ammonium dihydrogen phosphate (NH_4_H_2_PO_4_, Sinopharm Chemical Reagent Co., Ltd.) were dissolved in deionized water, and the Mg(NO_3_)_2_·6H_2_O solution was added dropwise into the NH_4_H_2_PO_4_ solutions with stirring, to produce a white precipitate. During the process, the pH was maintained at approximately 9 using an NH_3_·H_2_O solution (Shanghai Lingfeng Chemical Reagent Co., Ltd.). After stirring for 12 hours, the precipitate was filtered, and washed with distilled water 3 times. The obtained precipitate was dried at 50 °C for 24 hours to afford the powder products. The as-prepared magnesium phosphate (MP0) powders were characterized using transmission electron microscopy (TEM, JEM2010, JEOL, Japan), energy dispersive spectroscopy (EDS, Falcon, USA), XRD (Geigerflex, Rigaku Co. Ltd., Japan), and FTIR spectroscopy (Magna-IR 550, Nicolet). The MP0 powders were placed into stainless steel moulds (Φ10 × 2 mm) under a pressure of 2 MPa for 3 min to produce the MP0 samples. The MP0 samples were sintered at 400 °C, 600 °C and 800 °C to afford the various MP samples (i.e., 400 °C:MP4, 600 °C:MP6 and 800 °C:MP8), and the MP4, MP6 and MP8 samples were characterized using XRD and FTIR. The surface morphology of the MP0, MP4, MP6 and MP8 samples was observed using SEM (S-4800N, Hitachi, Japan).

### Degradability of MP in PBS

The *in vitro* degradability of the samples (Φ10 × mm) was assessed by measuring the weight loss ratio of the samples (i.e., MP0, MP4, MP6 and MP8) in phosphate buffered saline (PBS)^35^. The samples were weighed (W_i_) and immersed in PBS (pH = 7.4) in sealed polyethylene bottles with a solid/liquid ratio of 0.2 g/20 mL, and the bottles were placed in an orbital shaker at 37 °C with constant shaking for 12 weeks. The PBS solution was refreshed once a week. At different time points (3, 7, 14, 21, 28, 42, 56, 70 and 84 days), the samples were removed from the solution and rinsed gently with deionized water. Then, the samples were dried at 50 °C for 24 hours, and the samples were weighed (W_f_). The weight loss of the samples in PBS was calculated according to the following equation:

$${\rm{Weight}}\,{\rm{loss}}\,( \% )=({{\rm{W}}}_{{\rm{i}}}-{{\rm{W}}}_{{\rm{f}}})/{{\rm{W}}}_{{\rm{i}}}\times 100$$

The pH value of the PBS solution containing the samples was measured using a pH meter (FE20K, Mettler Toledo, Switzerland) at different time points (1, 2, 4, 12 and 21 days), and the surface morphology the samples was observed using SEM after soaking into the PBS solution for 3 weeks.

### Mineralization of MP in SBF

*In vitro* mineralization of the MP0, MP4, MP6 and MP8 samples (Φ10 × 2 mm) was determined after they were soaked in SBF (pH = 7.4) for different time periods (1, 2, 3, 5 and 7 days)^36^. At each time point, the samples were removed from the solution, and rinsed with deionized water. After drying in an oven at 37 °C for 24 h, the surfaces of the samples that were soaked in SBF for 7 days were characterized using SEM, EDS (Falcon, USA) and XRD. Moreover, the changes in the Mg, Ca and P ion concentrations in the solution after the samples were soaked in SBF for different time periods were determined by ICP-AES (IRIS 1000, Thermo Elemental, USA).

### Cell morphology on MP

The MG63 cells (ATCC; Chinese Academy of Sciences, Shanghai, China) were cultured in Dulbecco’s Modified Eagle Medium (DMEM; Hyclone, Thermo Fisher Scientific Inc., MA, USA) supplemented with 10% fetal bovine serum (FBS; GibcoBRL, Grand Island, NY, USA), 1% penicillin (100 U/mL) and streptomycin sulfate (100 mg/mL) (GibcoBRL, Grand Island, NY, USA). The cells were cultured at 37 °C in a humidified atmosphere consisting of 5% CO_2_ in air, and the culture medium was replaced every other day. The MP0, MP4, MP6 and MP8 samples (Φ10 × 2 mm) were placed into the wells of a 24-well plate (Costar, Corning Incorporated, NY, USA), and the cells were seeded in each well at a density of 3 × 10^4^/cm^2^. After the cells were seeded and cultured with the samples for 1, 3 and 5 days, the cells were fixed with 2.5% glutaraldehyde for 15 min and washed 3 times with PBS. Gradient ethanol with a volume fraction consisting of 30%, 50%, 70%, 90%, and 100% was used for 10 min to dehydrate the cells, and hexamethyldisilazane (HMDS, Sigma-Aldrich) was used to replace the ethanol. Finally, the samples were air-dried and observed via SEM.

### Cell proliferation on MP

The cell proliferation was determined using the CCK-8 assay at 1, 3 and 5 days after seeding according to a standard procedure. The MP0, MP4, MP6 and MP8 specimens (Φ10 × 2 mm) were sonicated in ethanol and sterilized under ultra-violet light. 1 mL of culture medium with 3 × 10^4^ MG63 cells was seeded on the samples, in 24-well tissue culture plates. At each time point, the samples were gently rinsed with PBS (×3) and then transferred to a new 24-well plate. 500 μL of DMEM and 50 μL of the CCK-8 solution (Dojindo Molecular Technologies Inc., Kumamoto, Japan) were added to each well, and the empty wells containing DMEM were used as a negative control. After 3 h, 100 μL of the supernatant was transferred into a 96-well plate and read at 450 nm using a microplate reader (Synergy HT, Bio-tek, Winooski, Vermont, USA) with 620 nm as the reference wavelength.

### ALP activity and osteogenic differentiation-related genes expression on MP

After incubation in a 24-well plate for 24 h, the culture medium was exchanged for an osteogenic induction medium (i.e., Minimum Essential Medium Alpha Modified culture medium), which was supplemented with 10% FBS, 0.1 μM dexamethasone (Sigma), 50 μM ascorbate acid (Sigma), and 10 μM β-glycerophosphate sodium (Sigma). These media were renewed every other day for 2 weeks. After 7, 10 and 14 days, the samples were washed with PBS (×3), and then lysed in a 0.2% TritonX-100 solution through four standard freeze-thaw cycles. ALP activity of cell lysates was normalized to the total protein content of each lysate.

The real-time polymerase chain reaction (PCR) was employed to quantitatively determine the mRNA expression of osteogenic differentiation-related genes, including mouse alkaline phosphatase (ALP), collagen type I (COL 1), osteoprotein (OPN) and osteocalcin (OC). Table S1 shows the sequences of the forward and reverse primers, and the genes transcription levels were normalized to the β-actin housekeeping gene. According to the manufacturer’s protocol, the total RNA was collected from the cells on the MP0, MP4, MP6 and MP8 samples using the Trizol reagent (Ambion, Grand Island, New York, USA). Reverse transcription was carried out following the protocol for the Revert-Aid first strand cDNA synthesis kit (Fermentas, Thermo Scientific Molecular Biology, Pittsburgh, Pennsylvania, USA). The SYBR premix EX TaqTM PCR kit (TaKaRa Biotechnology Co., Dalian, China) was used for quantitative testing in real-time PCR.

### Statistical analysis

All quantitative data, which are expressed as the mean ± standard deviation (M ± SD), were analyzed with Origin 8.0 (Origin Lab Corporation, USA). Statistical comparisons were carried out using analyses of variance (ANOVA). Statistical significance was achieved with a confidence level greater than 95% (p < 0.05).

## Electronic supplementary material

Dataset 1

## References

[1] Symietz C (2011). Fixation of bioactive calcium alkali phosphate on Ti6Al4V implant material with femtosecond laser pulses. Appl. Surf. Sci..

[2] Neel EAA (2009). Bioactive functional materials: a perspective on phosphate-based glasses. J. Mater. Chem..

[3] Renno ACM (2013). Incorporation of bioactive glass in calcium phosphate cement: Material characterization and *in vitro* degradation. J. Biomed. Mater. Res. A.

[4] Gao P (2016). Beta-tricalcium phosphate granules improve osteogenesis *in vitro* and establish innovative osteo-regenerators for bone tissue engineering *in vivo*. Sci. Rep.-UK.

[5] Xiao X (2015). The promotion of angiogenesis induced by three-dimensional porous beta-tricalcium phosphate scaffold with different interconnection sizes via activation of PI3K/Akt pathways. Sci. Rep.-UK.

[6] Zhang J (2015). Magnesium modification of a calcium phosphate cement alters bone marrow stromal cell behavior via an integrin-mediated mechanism. Biomaterials.

[7] Wong HM (2013). *In vivo* stimulation of bone formation by aluminum and oxygen plasma surface-modified magnesium implants. Biomaterials.

[8] Staiger MP, Pietak AM, Huadmai J, Dias G (2006). Magnesium and its alloys as orthopedic biomaterials: A review. Biomaterials.

[9] Witte F (2007). Biodegradable magnesium-hydroxyapatite metal matrix composites. Biomaterials.

[10] Huang BL (2016). Facilitated receptor-recognition and enhanced bioactivity of bone morphogenetic protein-2 on magnesium-substituted hydroxyapatite surface. Sci. Rep.-UK.

[11] Yoshizawa S, Chaya A, Verdelis K, Bilodeau EA, Sfeir C (2015). An *in vivo* model to assess magnesium alloys and their biological effect on human bone marrow stromal cells. Acta Biomater..

[12] Liu WJ, Zhai D, Huan ZG, Wu CT, Chang J (2015). Novel tricalcium silicate/magnesium phosphate composite bone cement having high compressive strength, *in vitro* bioactivity and cytocompatibility. Acta Biomater..

[13] Wu F, Liu CS, O’Neill B, Wei J, Ngothai Y (2012). Fabrication and properties of porous scaffold of magnesium phosphate/polycaprolactone biocomposite for bone tissue engineering. Appl. Surf. Sci..

[14] Wu F, Su JC, Wei J, Guo H, Liu CS (2008). Injectable bioactive calcium-magnesium phosphate cement for bone regeneration. Biomed. Mater..

[15] Wu F (2008). Self-setting bioactive calcium-magnesium phosphate cement with high strength and degradability for bone regeneration. Acta Biomater..

[16] Ostrowski N, Roy A, Kumta PN (2016). Magnesium Phosphate Cement Systems for Hard Tissue Applications: A Review. ACS Biomater. Sci. Eng.

[17] Babaie E, Lin B, Goel VK, Bhaduri SB (2016). Evaluation of amorphous magnesium phosphate (AMP) based non-exothermic orthopedic cements. Biomed. Mater..

[18] Meininger M (2016). Electrochemically assisted deposition of strontium modified magnesium phosphate on titanium surfaces. Mat. Sci. Eng. C-mater.

[19] Lee J, Farag MM, Park EK, Lim J, Yun HS (2014). A simultaneous process of 3D magnesium phosphate scaffold fabrication and bioactive substance loading for hard tissue regeneration. Mat. Sci. Eng. C-mater.

[20] Meininger S (2016). Strength reliability and *in vitro* degradation of three-dimensional powder printed strontium-substituted magnesium phosphate scaffolds. Acta Biomater..

[21] Zhou H, Luchini TJ, Bhaduri SB (2012). Microwave assisted synthesis of amorphous magnesium phosphate nanospheres. J. Mater. Sci.-mater. M.

[22] Zhou H, Nabiyouni M, Lin BR, Bhaduri SB (2013). Fabrication of novel poly(lactic acid)/amorphous magnesium phosphate bionanocomposite fibers for tissue engineering applications via electrospinning. Mat. Sci. Eng. C-mater.

[23] Qi C, Zhu YJ, Chen F, Wu J (2015). Porous microspheres of magnesium whitlockite and amorphous calcium magnesium phosphate: microwave-assisted rapid synthesis using creatine phosphate, and application in drug delivery. J. Mater. Chem. B.

[24] Qi C (2016). Magnesium phosphate pentahydrate nanosheets: Microwave-hydrothermal rapid synthesis using creatine phosphate as an organic phosphorus source and application in protein adsorption. J. Colloid Interf. Sci.

[25] Nabiyouni M, Ren YF, Bhaduri SB (2015). Magnesium substitution in the structure of orthopedic nanoparticles: A comparison between amorphous magnesium phosphates, calcium magnesium phosphates, and hydroxyapatites. Mat. Sci. Eng. C-mater.

[26] Sun M (2016). Systematical Evaluation of Mechanically Strong 3D Printed Diluted magnesium Doping Wollastonite Scaffolds on Osteogenic Capacity in Rabbit Calvarial Defects. Sci. Rep.-UK.

[27] Comesana R (2015). Toward Smart Implant Synthesis: Bonding Bioceramics of Different Resorbability to Match Bone Growth Rates. Sci. Rep.-UK.

[28] Sheikh Z (2015). Mechanisms of *in Vivo* Degradation and Resorption of Calcium Phosphate Based Biomaterials. Mater.

[29] Gao X (2016). Polydopamine-Templated Hydroxyapatite Reinforced Polycaprolactone Composite Nanofibers with Enhanced Cytocompatibility and Osteogenesis for Bone Tissue Engineering. ACS Appl. Mater. Inter..

[30] Choi S (2013). Inorganic coatings for optimized non-viral transfection of stem cells. Sci. Rep.-UK.

[31] Heimann RB, Wirth R (2006). Formation and transformation of amorphous calcium phosphates on titanium alloy surfaces during atmospheric plasma spraying and their subsequent *in vitro* performance. Biomaterials.

[32] Zhang J (2015). Magnesium modification of a calcium phosphate cement alters bone marrow stromal cell behavior via an integrin-mediated mechanism. Biomaterials.

[33] Toita R, Tsuru K, Ishikawa K (2016). Immobilization of calcium and phosphate ions improves the osteoconductivity of titanium implants. Mat. Sci. Eng. C-mater.

[34] Puvaneswary S (2016). Incorporation of Fucoidan in beta-Tricalcium phosphate-Chitosan scaffold prompts the differentiation of human bone marrow stromal cells into osteogenic lineage. Sci. Rep.-UK.

[35] Yoshioka T, Kawazoe N, Tateishi T, Chen G (2008). *In vitro* evaluation of biodegradation of poly(lactic-co-glycolic acid) sponges. Biomaterials.

[36] Neel EEA (2007). *In vitro* bioactivity and gene expression by cells cultured on titanium dioxide doped phosphate-based glasses. Biomaterials.

