# Supplementary material for: Effects of sintering temperature on surface morphology/microstructure, in vitro degradability, mineralization and osteoblast response to magnesium phosphate as biomedical material
Source: Sci Rep. 2017 Apr 11;7:823. doi: 10.1038/s41598-017-00905-2 (PMC5429756; doi:10.1038/s41598-017-00905-2)
Supplement: Supplementary file 1 — Dataset 1 [file 41598_2017_905_MOESM1_ESM.doc]

**Effects of sintering temperature on surface morphology/microstructure, in vitro degradability, mineralization and osteoblast response to magnesium phosphate as biomedical material**

Zhiwei Wang^1^, Yuhai Ma^1^, Jie Wei^2^, Xiao Chen^1^, Liehu Cao^1^, Weizong Weng^1^, Quan Li^1^, Han Guo^3^, and Jiacan Su^1,*^

^1^Department of Orthopaedics, Changhai Hospital, Second Military Medical University, Shanghai 200433, China

^2^Key Laboratory for Ultrafine Materials of Ministry of Education, East China University of Science and Technology, Shanghai 200237, China

^3^Shanghai Synchrotron Radiation Facility, Shanghai Institute of Applied Physics, Chinese Academy of Sciences, Shanghai 201800, PR China

*Corresponding author: e-mail:

Tel.: +86-21-81873400; Fax: +86-21-81873398

**Supporting information**

Supplementary Table 1 Primers for Real-time PCR

| Target genes | Primer sequences (5’-3’) |
| --- | --- |
| ALP | F:GGGCATTGTGACTACCACTCG |
|  | R:CCTCTGGTGGCATCTCGTTAT |
| COL 1 | F:AACAGTCGCTTCACCTACAGC |
|  | R:GGTCTTGGTGGTTTTGTATTCG |
| OC | F:GGACCATCTTTCTGCTCACTCTG |
|  | R:TTCACTACCTTATTGCCCTCCTG |
| β-actin | F:GAGACCTTCAACACCCCAGC |
|  | R:ATGTCACGCACGATTTCCC |

Supplementary Table 2 The Mg, P and Ca ion concentrations in SBF for MP0, MP4, MP6 and MP8 after soaking for 7 days

| Samples | Mg (mg/L) | P (mg/L) | Ca (mg/L) |
| --- | --- | --- | --- |
| MP0 | 502 | 325 | 82 |
| MP4 | 436 | 289 | 58 |
| MP6 | 365 | 260 | 24 |
| MP8 | 313 | 247 | 8.5 |
